# Content of lead and cadmium in aboveground plant organs of grasses growing on the areas adjacent to a route of big traffic

**DOI:** 10.1007/s11356-014-3634-9

**Published:** 2014-10-08

**Authors:** K. Jankowski, A. G. Ciepiela, J. Jankowska, W. Szulc, R. Kolczarek, J. Sosnowski, B. Wiśniewska-Kadżajan, E. Malinowska, E. Radzka, W. Czeluściński, J. Deska

**Affiliations:** 1 Siedlce University of Natural Sciences and Humanities, ul. B. Prusa 14, 08-110 Siedlce, Poland; 2 Warsaw University of Life Sciences, Warsaw, Poland

**Keywords:** Heavy metals, Inflorescence, Distance from, Pollution source, Road, Deposition

## Abstract

The effect of traffic on the content of lead and cadmium in grass morphological parts—leaves, shoots, and inflorescences—was studied. The samples were taken on a part of the European route E30 (Siedlce by road). The following plants were tested: *Dactylis glomerata*, *Arrhenatherum elatius*, and *Alopecurus pratensis*. During the flowering of grasses, the plant material was collected at distances of 1, 5, 10, and 15 m from the edge of the road, on the strip of road with a length of 9 km. In the collected plant parts, the content of lead and cadmium using the atomic absorption spectroscopy (AAS) method was determined. The effect of distance from the road on the content of lead and cadmium was evaluated using regression equations. Average lead content in the above parts of tested grass species was 3.56, while cadmium 0.307 mg kg^−1^ dry matter (DM). Lead content in plants of *Alopecurus pratensis* (average 4.11 mg kg^−1^ DM) was significantly higher than in other grasses. The lowest cadmium content, significantly different from the other species, was found in plants of *Arrhenatherum elatius* (0.251 mg kg^−1^ DM). Distance of sampling sites from the roadway significantly affects the differences in the content of cadmium and lead in plants. Analyzed aboveground plant organs of studied grasses were significantly different in contents of lead and cadmium. There were species differences in the proportions of cadmium concentration in various organs of plants. The obtained results indicate the possibility of species composition selection of grassland sward in areas with a higher risk of heavy metals associated with dust sedimentation.

## Introduction

Grasses are typical cover of soils adjacent to the roadway. They are subjected to continuous operation of stressful factors caused by the exploitation of the roads. These are mainly emissions, dust from the wear of tires and the clutch disc, and the chemical substances used to maintain the roads in the winter (Abollino et al. 2002; Conde et al. 2009; Jiries et al. 2002; Petrotou et al. 2012). Motor vehicles introduce a number of pollutants into the environment. Roadsides receive considerable amounts of these traffic-generated pollutants (Garcia and Milan 1998). The effect of these factors is the change of physical and chemical conditions of their growth and bioaccumulation of elements deposited into the environment. Among the many chemicals deposited during road use, the greatest impact is brought by heavy metals, including lead and cadmium. Existing in the soil, large quantities of these elements and fresh emission affect the increase of their content in the plants growing along the road. Their content in the plants is, however, mainly related with bioaccumulation potential specific for each species. The occurence of high bioaccumulation of heavy metals in plants has been described in many publications (Burt et al. 2003; Dai et al. 2004; Donisa et al. 2000; Mulchi at al. 2011; Schwarth et al. 1999; Samecka-Cymerman et al. 2009; Smith et al. 1990; Vasu et al. 1998). By constant changes of the properties of fuels and vehicle construction materials, we can observe that a harmful impact of transport on the environment is still modifying (Viard et al. 2004). However, large amounts of heavy metals accumulated over the years prove their permanent hazardous effect on plant quality (Petrotou et al. 2012). A ban on fuels containing lead has reduced their emissions into the atmosphere and consequently improved the condition of biota adjacent to a road. However, still possible are heavy metal emissions with the dust coming from the wearing parts of working vehicles. It has resulted in the need to control the level of assessment of heavy metal accumulation in organisms inhabiting areas adjacent to the roads. Their content in the plants is, however, mainly connected with bioaccumulation potential specific for each species (Aoyama and Kuroyanagis 1996; Bulinski et al. 1990; Derome and Lindroos 1998; Gaw et al. 2006; Hildebrandt et al. 2009; Naszradi et al. 2004). Accumulation of dust with fine particles of heavy metals in aboveground parts of the plants has a direct impact on contamination of plants growing in their anthropogenic conditions (Hendry 1992; Jiries 2002; Parekh et al. 1990). The ability of plants to a diverse accumulation of heavy metals in aboveground part is due to different morphology of plants (Deska et al. 2011). A major role in this process play the structure occurring on the surfaces of leaves—grooves, hair, and bristles—or specific chemicals—wax and others (Naszradi et al. 2004). They cause a variety of options to keep the dust on the surface of plants. It may also be the differences in accumulation of heavy metals in the different parts of the species (Stafilov and Jordanovska 1997). It is sometimes caused by genetics, morphology, or the length of biosorption (Dudka et al. 1983; Naszradi et al. 2004; Parekh et al. 1990; Viard et al. 2004). The most common are the differences between the content of these elements in the leaves and seeds of plants (Banuelos and Ajwa 1999; Dudka et al. 1983). They are also often seen as interspecific differences in bioaccumulation of heavy metals (Viard et al. 2004). Content of heavy metals near the roads can cause both different pollution of grass species and their morphological parts (Naszradi et al. 2004; De Nicola et al. 2003). The aims of the study were to evaluate the influence of distance from the road on the content of lead and cadmium in aboveground parts of three grass species near a fast road and to assess bioaccumulation of these elements by morphological parts of the grasses.

## Material and methods

**Fig. 1:**
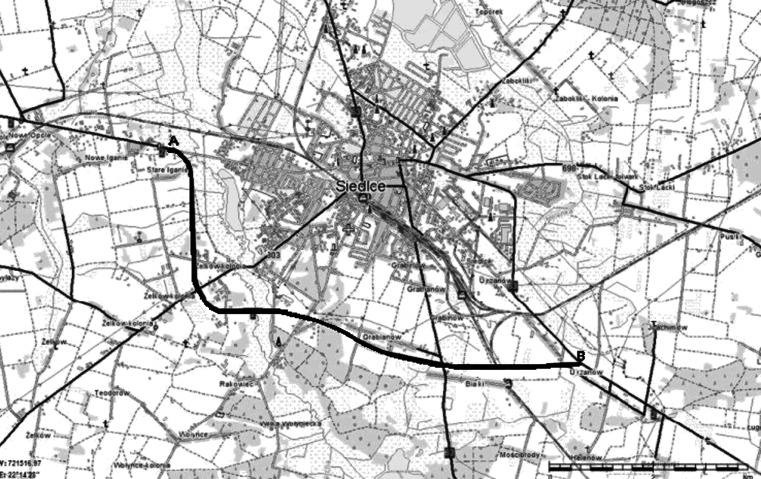
Schematic presentation of the studied sites of S2 Road

The plant materials in the form of the aboveground parts of three grasses were taken in August 2011 along a 9-km sector of the speed roadway S2 (Siedlce Bypass) (Fig. 1). The track connects Cork in Ireland with Omsk in Russia and belongs to the most important tracks of communication in Europe, and in Poland, it runs along the motorway S2. The area, where samples were collected, belongs to the Mazovian voivodeship, which is located in the middle-east of Poland about 80 km on the east from Warsaw. Weather conditions of research area were typical for IX - eastern district of agro-climatic of Poland. Average annual air temperature ranges from 6.7 to 6.9 °C, and in summer, the average daily temperature is 15 °C. Annual precipitation is at the level 550–650 mm, while they are not frequent, but heavy. In 2011 in May, June, August, and September by Selianinov method were poor drought (Bac et al. 1993). Average daily movement of motor vehicles (SDR) in the year of study was 9,888 vehicles per day, whereas on the remaining national roads 7,097 engines per day, and in case of international roads 16,667 per day. On the analyzed section of road (ring road for Siedlce), the average daily movement of motor vehicles was higher than the average on national roads of Poland, and it was 8,136 vehicles per day.

Chosen species were collected from the grasslands located near the international rout. The following plants were tested: *Dactylis glomerata*, *Arrhenatherum elatius*, and *Alopecurus pratensis. D. glomerata* grows in dense perennial tussocks to 150 cm tall, with leaves of 20–50 cm long and up to 1.5 cm broad, and a distinctive tufted triangular flower head of 10–15 cm long. *Arrhenatherum elatius* is a loosely tufted, deciduous, perennial grass with upright to arching, broadly linear, slightly hairy, bright green leaves and erect stems bearing green to purple flower from early summer into autumn. *Alopecurus pratensis* is a long-lived, tufted perennial grass, with short rhizomes and short ascending stolons, loose or compact tufts, erect culms of 30–100 cm tall, and flat, narrow, 2–6 mm broad, glaucous, glabrous leaf blades. The plant material (a total number of 60 samples) was taken from distances of 1, 5, 10, and 15 m from both the sides of the road at intervals of approximately 100 m. Individual samples were collected as morphological parts of plants—leaves, shoots, and inflorescences. From the individual samples, five samples for each species of plants and any distance from the edge of the road were taken. The total sampling amounted to 180 (5 samples for each species = 15 × 4 different distances × 3 repetitions).

Collected plant samples (not washed) were dried in the temperature of 105 °C, weighed and dry mineralized in the temperature of 450 °C for 24 h, and then prepared in 10 % volume HCL (Viard, et al. 2004). Content of lead and cadmium has been marked in accordance with atomic absorption spectroscopy (AAS) method with the use of AAS instrument (Thermo Elemental, M6 Solar type, Cambridge UK company).

To verify the accuracy of the analytical models, internal quality control patterns were used. It was assumed that the average recovery of tested patterns should range from 85 to 115 % of actual value. For research needs, two quality control standards were prepared. Their analysis was carried out in each series of samples. The obtained recovery was contaminated within a given range.

All data in this work were expressed as means ± standard deviation (SD). The collected data were analyzed statistically using the Statistica 10.0. The mean arithmetic and coefficients of variation (V) were calculated. Effects of tested factors (grass species, plant part, the distance from the road) on the content of elements in plant were estimated using three-way analysis of variance. Detailed comparisons of the mean values were based on Tuckey’s test at *p* ≤ 0.05. In the case of quantitative impact factor (distance), the nature of this impact examined was analyzed using the orthogonal contrasts. To assess the relationship between the content of lead and cadmium in morphological parts of the studied grass species and distance from the road, we used a linear regression method, using polynomial second degree:

$$ {Y}_{\mathrm{i}}={a}_1{x}^2+{a}_2x+{a}_0 $$

where *Y*_i_ is the depended variable (metal content), *x* is the explanatory variable (the distance from the road), *a*_0_ is the intercept, and *a*_2_ is the regression coefficient, indicating how much the change of the size of the depended variable (*Y*); when the independent variable increased about 1 unit, the other values were stable. For each equation, the coefficient of determination (*R* ^2^) was estimated which indicated which part of the total variation with *Y* variable explained the regression model (Deska et al. 2011).

## Results and discussion

### Differences of Pb and Cd concentrations between the three species

Average lead content in the aboveground parts of tested grass species amounted to 3.56 mg kg^−1^ dry matter (DM; Table 1). In *Alopecurus pratensis* plants, significantly greater amounts of this element (an average of 4.11 mg kg^−1^ DM) than in the other studied grass species were stated. It has showed a statistical analysis.

**Table 1:** The content of lead and cadmium in samples of plants: *Dactylis glomerata*, *Arrhenatherum elatius*, and *Alopecurus pratensis* (mg kg^−1^)

Species of grass	Lead $\overline{x}$	Cadmium $\overline{x}$
*Dactylis glomerata*	3.27 ± 0.62a	0.345 ± 0.02b
*Arrhenatherum elatius*	3.30 ± 0.61a	0.251 ± 0.01a
*Alopecurus pratensis*	4.11 ± 0.52b	0.324 ± 0.06b
An average for the species	3.56 ± 0.27	0.307 ± 0.02

Values marked with the same letter are not significantly different in the columns at *p* ≤ 0.05

The lead content found in different grass species from a variety parts of Poland reported by Klocek et al. (2003) ranged from 0.6 to 15 mg kg^−1^ DM. The average value reported by these authors is 2.5 mg kg^−1^ DM. In other countries (Donisa et al. 2000; Petrotou et al. 2012), it was found that the average size of this element concentration in the grasses was from 0.4 (Greece) to 4.6 mg kg^−1^ DM (Slovakia). High uptaking of this element is recorded in nearby communication routes, because the lead uptake by plants is greater in the presence of organic compounds existing in the combustion products (Naszradi et al. 2004; Viard et al. 2004). By Deska et al. (2011), the lead content in the soil adjacent to the road on the study section ranged from 91.3 to 101.6 mg kg^−1^ DM, which is affected on the higher level of their absorption in plants. Having compared the obtained results with a limit of lead content in feed for ruminants (according to Klocek et al. (2003), these amount to 10 mg kg^−1^ DM), we can observe that the tested grasses are classified for use in the animal feeding. It should also be noted that the analyzed species of grasses have a healthy appearance, because their content has not reached the level of lead toxicity (Bulinski et al. 1990). Plant poisoning with lead, according to Klocek and Milczarek (2003), may occur when the content is more than 15 mg of this element per kilogram of dry matter. This amount of lead can impair the process of photosynthesis by reducing chlorophyll biosynthesis (Parekh et al. 1990; Viard et al. 2004). With regard to Pb, the main roadside pollutant concentrations found in these studies are much less than those reported by other studies (Mulchi et al. 2001; Garcia and Milan 1998).

Another significantly important metal in plants growing on the areas adjacent to the speed roadway is cadmium; Klocek et al. (2003) claim that critical cadmium content in plants in relation to their usefulness for consumptive aims should not exceed the value of 0.15 mg kg^−1^ DM, but for fodder aims, it should come to ≤0.5 mg kg^−1^ DM. Cadmium content in the studied grass species samples was much lower and ranged from 0.251 to 0.345 mg kg^−1^ DM (Table 1). Statistical analysis showed a significant effect of grass species on the content of this element. The lowest, significantly different from the other species, was the content of this element found in samples of *Arrhenatherum elatius*. Deska et al. (2011) studied an assessment of the cadmium content in the sward of grass species and found species diversity of this element content. In Poland, the content of this element in various grass species ranged from 0.1 to 2.6 mg kg^−1^ DM (Klocek et al. 2003). In northeastern Poland, cadmium content in plants of *D. glomerata* ranged from 0.05 to 0.80 g kg^−1^, as well as in the southwestern region from 0.10 to 2.60 g kg^−1^. While, cadmium content in the grasses found in other studies was in the range of 1.0–1.6 mg kg^−1^ DM for Belgium (Viard et al. 2004) and 0.3–2.9 mg kg^−1^ DM for Hungary (Naszradi et al. 2004).

As in the case of other heavy metals, cadmium content in plants depends on the intensity of the dust emission containing this element and on the concentration of cadmium in soil and on physico-chemical parameters of the soil (Abollino et al. 2002; Li et al. 2008; Petrotou et al. 2012). The cadmium content in soils adjacent to this roadway was in the range from 0.195 to 0.303 mg kg^−1^ DM (Deska et al. 2011).

### Differences of Pb and Cd concentrations between different plant organs

The type of morphological part of tested plant significantly differentiated the lead content in different grass species (Table 2). Significantly lower amounts of lead than in the other organs were stated in the leaves of grass species—2.82 mg kg^−1^ DM. In the case of *D. glomerata* and *Arrhenatherum elatius*, lead content was distributed in a similar way. The smallest amount, significantly lower than in the other morphological parts, was determined in the leaves of these plants—2.35 and 2.70 mg kg^−1^ DM). Average lead content in the leaves of *D. glomerata* was the lowest from the all parts of the studied grass species. In the case of *Alopecurus pratensis*, lead content differed significantly in inflorescences of plants—5.04 mg kg^−1^ DM. It was the highest average from the obtained results. Cadmium content distributed dissimilarly (Table 2). The highest, significantly different from the rest of cadmium values, was found in the leaves of grasses—0.387 mg kg^−1^ DM. In *D. glomerata*, the content of this element in the inflorescences was significantly lower (0.221 mg kg^−1^ DM) than in other organs of the studied species. Morphological parts of *Arrhenatherum elatius* did not differ from each other in cadmium content. In the case of *Alopecurus pratensis*, all parts of the tested plants had significantly different concentrations of this element; the least was in the shoots—0.162 mg kg^−1^ DM—and the most in the leaves—0.485 mg kg^−1^ DM.

**Table 2:** The content of lead and cadmium in samples of plants: *Dactylis glomerata*, *Arrhenatherum elatius*, and *Alopecurus pratensis* (mg kg^−1^)

Species of grass	Some morphological	Lead $\overline{x}$	Cadmium $\overline{x}$
*Dactylis glomerata*	Leaves	2.35 ± 0.67a	0.466 ± 0.036b
	Inflorescence	4.02 ± 0.44b	0.221 ± 0.074a
	Shoot	3.43 ± 0.61b	0.368 ± 0.040b
*Arrhenatherum elatius*	Leaves	2.70 ± 0.75a	0.230 ± 0.039a
	Inflorescence	3.54 ± 0.11b	0.237 ± 0.089a
	Shoot	3.65 ± 0.76b	0.285 ± 0.045a
*Alopecurus pratensis*	Leaves	3.40 ± 0.52a	0.485 ± 0.013c
	Inflorescence	5.04 ± 0.35b	0.325 ± 0.088b
	Shot	3.90 ± 0.25a	0.162 ± 0.058a
An average for the species	Leaves	2.82 ± 0.13a	0.387 ± 0.058b
	Inflorescence	4.20 ± 0.65b	0.261 ± 0.049a
	Shoot	3.65 ± 0.46b	0.272 ± 0.093a

Values marked with the same letter do not differ significantly in columns within each species at *p* ≤ 0.05

### Effects of the distance from the road on of Pb and Cd concentrations

The distance from the road in different ways influences on the content of the analyzed elements in described grasses (Table 3). The largest amounts of lead in plants (4.39 mg kg^−1^ DM), significantly different from the others, were found in plants growing within 5 m from the edge of the road. This value differed significantly from the lead content in the plant material defined at the other distances from the road. Also, in the study of Yusuf et al. (2003), lead exhibited a gradual decrease from 0- to 15-m distance. In the case of cadmium, the largest amount (significantly different from the others) was stated in plants grown in the distances of 1 and 5 m from the road—0.380 and 0.377 mg kg^−1^ DM. Similar correlations of lead content on the distance from the roadway were found for *D. glomerata* and *Arrhenatherum elatius*. The highest, significantly different, lead content was found in samples taken at a distance of 5 m from the road: an average of 4.72 and 5.08 mg kg^−1^ DM. The lowest, significantly different from the other trials, was found in samples of plants from distances of 1 and 15 m—2.73 and 2.20 mg kg^−1^ DM (*D. glomerata*), and 2.52 and 1.64 mg kg^−1^ DM (*Arrhenatherum elatius*). In the case of *Alopecurus pratensis*, the smallest amounts of lead, significantly different, were found for plants at a distance of 1 m—3.13 mg kg^−1^ DM—and the largest, also significantly different, from a distance of 15 m—5.18 mg kg^−1^ DM.

**Table 3:** The content of lead and cadmium in plants *Dactylis glomerata*, *Arrhenatherum elatius*, and *Alopecurus pratensis* (mg kg^−1^) depending on the distance from the road

Distance from the road (m)	Plant species	Lead	Cadmium
		Mean $\overline{x}$	Mean $\overline{x}$
1	*Dactylis glomerata* *Arrhenatherum elatius* *Alopecurus pratensis*	2.73 ± 0.35aA 2.52 ± 0.41aA 3.13 ± 0.35aA	0.428 ± 0.050bC 0.292 ± 0.018aB 0.337 ± 0.018aA
Average for 1 m		3.02 ± 0.78A	0.380 ± 0.020B
5	*Dactylis glomerata* *Arrhenatherum elatius* *Alopecurus pratensis*	4.72 ± 0.60bC 5.08 ± 0.10bC 4.09 ± 0.27aB	0.447 ± 0.020cC 0.272 ± 0.061aB 0.372 ± 0.014bA
Average for 5 m		4.39 ± 0.80B	0.377 ± 0.017B
10	*Dactylis glomerata* *Arrhenatherum elatius* *Alopecurus pratensis*	3.41 ± 0.26aB 3.96 ± 0.57aB 4.02 ± 0.82aB	0.287 ± 0.013aB 0.327 ± 0.014aB 0.285 ± 0.012aA
Average for 10 m		3.52 ± 0.70A	0.283 ± 0.013A
15	*Dactylis glomerata* *Arrhenatherum elatius* *Alopecurus pratensis*	2.20 ± 0.90aA 1.64 ± 0.60aA 5.18 ± 0.80bC	0.187 ± 0.053bA 0.111 ± 0.036aA 0.303 ± 0.013cA
Average for 15 m		3.01 ± 0.97A	0.200 ± 0.011A

Values marked by the same small letters do not significantly differ in the content of elements in different species for the same distance, at *p* ≤ 0.05. Values marked with the same capital letters do not significantly differ in the content of elements in plants of one species at different distances, at *p* ≤ 0.05

The lead content in different samples of the grass species growing at 1 and 10 m from the road did not differ significantly (Table 3). In case of other distances, *Alopecurus pratensis* reacted in a different way. At a distance of 5 m, plants of this species contained the highest amounts of this element, and at distance of 15 m the smallest.

In Viard et al. (2004), a study on the impact of traffic on the content of heavy metals in the grass of *Festuca arundinacea*, *Phallaris* sp*.*, and *D. glomerata* gathered beside the road, the lead contents amounted to 1.0–2.0 mg kg^−1^ DM, but growing at 5 and 20 m from the road—0.8–2.2 and 0.5–0.7 mg kg^−1^ DM. Distance from the road significantly affects the cadmium content in different studied grass species (Table 3). In case of *D. glomerata*, cadmium content was the highest, significantly different from the other species. At distances of 1 and 5 m from the road, it amounted on average to 0.428 and 0.447 mg kg^−1^ DM. The lowest amounts of the element, also significantly different, were found at a distance of 15 m—0.187 mg kg^−1^ DM. *Arrhenatherum elatius* contained significantly different amounts of cadmium, lower than in case of the other species, and they reached values, at 15 m, of 0.111 mg kg^−1^ DM. *Alopecurus pratensis* proved no significant influence of distance from the road on bioaccumulation of cadmium by the plant.

At 1-m distance from the edge of the road, the greatest amounts of cadmium were found in samples of *D. glomerata* (0.428 mg kg^−1^). Additionally, at 5-m distance, the highest cadmium content was observed in the samples of *D. glomerata* (0.447 mg kg^−1^), and the lowest in *Arrhenatherum elatius* (0.272 mg kg^−1^). These amounts differ from the metal content in *Alopecurus pratensis* (0.372 mg kg^−1^). At a distance of 10 m, cadmium content in various grass species did not differ. At a distance of 15 m, the smallest amount of this element, significantly different, was found in *Arrhentherum elatius* (0.111 mg kg^−1^), and the highest, also significantly different, in *Alopecurus pratensis* (0.303 mg kg^−1^).

**Fig. 2:**
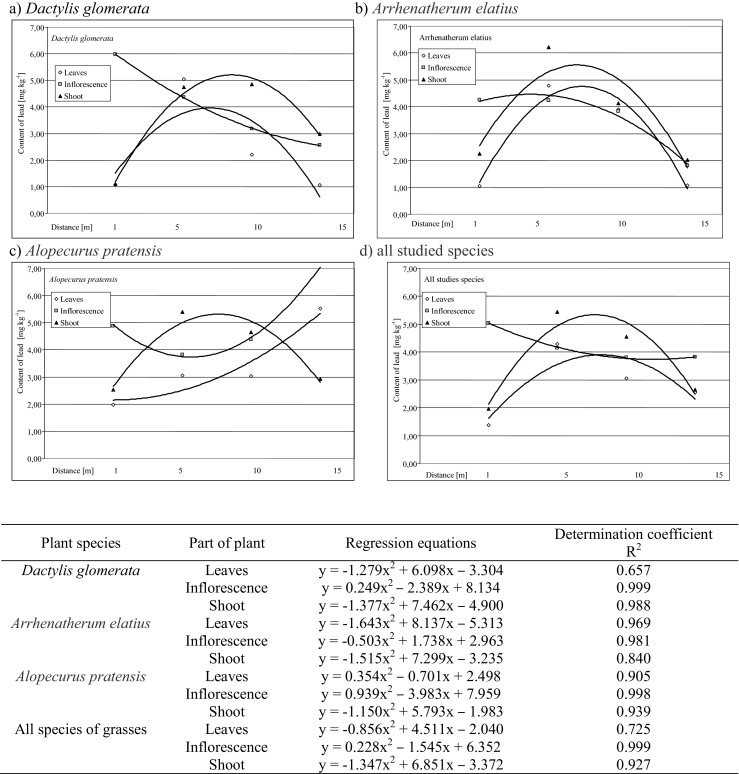
Course of regression equations describing the effect of distance from the road on the lead content in morphological parts of the studied grass species (*p* ≤ 0.05)

Regression equations describing the effect of the distance from the roadway on the lead content in the tested morphological parts of the grasses indicate high differences of accumulation of this element in the organs of the same species (Fig. 2). The most variation in the contents of heavy metals has been demonstrated in plants organs for *D. glomerata* and *Alopecurus pratensis*.

In the case of *D. glomerata* (Fig. 2a), the variability of lead content in the inflorescence showed an almost linear course, with the maximum value (6.0 mg kg^−1^ DM) at a distance of 1 m from the edge of the road. Variability of the lead content in the other organs of plants has been described by characteristic equations (Jiries et al. 2002; Deska et al. 2011) for this type of phenomena with a maximum at a distance of 7–8 m from the road. Such a high lead content in inflorescence of *D. glomerata* may be due to its specific construction, permitting dust keeping (Klocek et al. 2003).

In plants of *Alopecurus pratensis* (Fig. 2c), different relationships between the distance from the road and the lead content for the inflorescences and shoots were stated. Regression equations indicate an increase of the lead content in these plant parts in farther distances from the road from 4.89 to 7.08 mg kg^−1^, and from 2.53 to 2.93 mg kg^−1^. The same results (calculated by regression coefficient) were obtained in studies of other authors for *Arrhenatherum elatius* in the case of increased traffic (Naszradi et al. 2004; Samecka-Cymerman et al. 2009).

Variation of the lead content depending on the distance was also found in similar morphological parts of various species (Fig. 2a, b, c). Only with respect to the leaf, the coefficients of regression equations indicate a high mutual similarity of these functions. In the grasses of *D. glomerata* and *Arrhenatherum elatius*, similar response to the pollution of roadway area was also related to the shoots of plants. Different courses in all described grass species had regression equations describing the effect of distance from the roadway to lead contamination of plants inflorescences.

The regression equations for the averages of all the grasses (Fig. 2d) show the different courses of the equation of lead absorption in the inflorescences, in comparison with the equations describing this phenomenon for the shoots and leaves.

High values of the determination coefficients (*R* ^2^) show a very good fit of the used polynomial function of the second degree to describe this occurence. It does not concern equation describing the impact of distance on the lead concentration in leaves of *D. glomerata*.

**Fig. 3:**
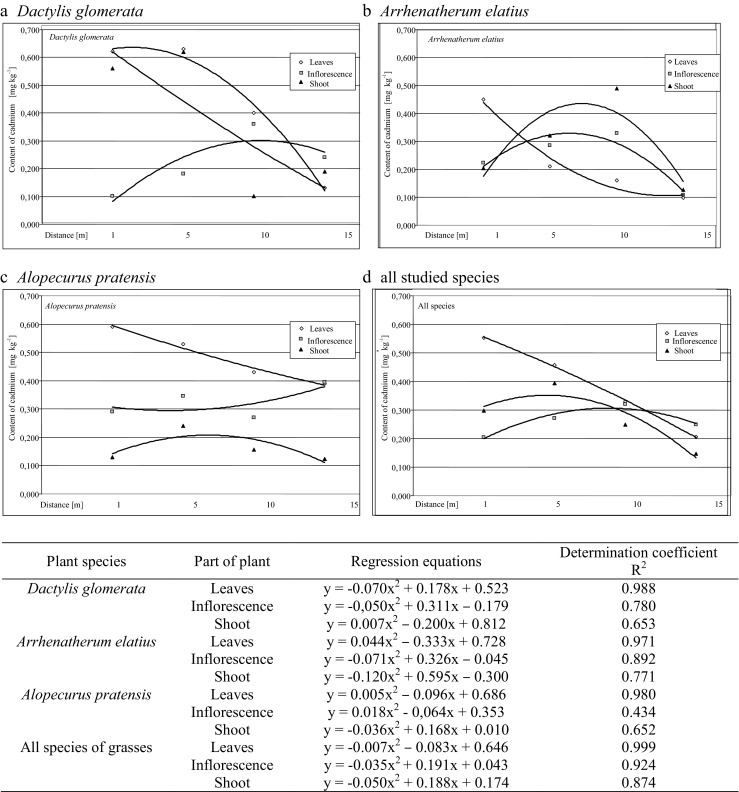
Course of regression equations describing the effect of distance from the road on the cadmium content in morphological parts of the studied grass species (*p* ≤ 0.05)

Different values than in case of lead shoved regression equation describing the affect of distance from the road on the amount of cadmium in aboveground plant organs (Fig. 3). The calculated ratios of these equations also show a great diversity in accumulation of cadmium in the organs of the same species. As mentioned above, the highest amounts of the element were found in *D. glomerata* and *Alopecurus pratensis*.

In *D. glomerata* (Fig. 3a), the highest content of cadmium in the strip adjacent to the roadway (1–5 m) was found in the leaves (0.621 and 0.630 mg kg^−1^ DM) and shoots (0.562 and 0.622 mg kg^−1^ DM). At the successive distances, the content of this metal in similar parts decreased, and for the shoots, this decline is virtually linear. This is evidenced by the low value of the coefficient *a*_1_ in the regression equation (Fig. 3). Variability of cadmium in the successive distances from the roadway in the inflorescences was described by parabolic equations, with a maximum content at distance of 10 m from the road (0.300 mg kg^−1^ DM).

The increase in the distance from the road induced different effects on the cadmium content in each described plant organs of *Alopecurus pratensis* (Fig. 3c). The largest amounts of cadmium in its leaves were found near the road lane (0.590 mg kg^−1^ DM). When the road distance increased, the content of this element almost linearly decreased (Fig. 3). Cadmium content in inflorescences increased from 0.290 (1 m) to 0.395 mg kg^−1^ DM (15 m), and in the shoots of this plant, the most content of this element was found in the middle of the tested strip (0.240 mg kg^−1^ DM).

Cadmium content in the leaves of *Arrhenatherum elatius* (Fig. 3b) was similar as in *Alopecurus pratensis*, but the relationship ‘distance–cadmium content’ is described by a less flattened curve (Fig. 3b). The highest content in shoots and inflorescences was found in plants taken from the strip of 5–10 m. Calculation for these coefficients of regression equations illustrates the typical response of heavy metals in plants to the occurence caused by the use of vehicles (Viard et al. 2004).

Regression equations describing the average for all species (Fig. 3d) show the different courses of cadmium absorption equation for tested grass leaves, in comparison to the shoots and leaves. High coefficients of determination (*R* ^2^) show a very good fit of the used polynomial functions to describe this phenomenon for the majority of reported cases. Slightly lower values of coefficient *R* ^2^ for just a very few cases indicate a smaller fit of polynomial function to describe this phenomenon, but this description is to be satisfactory (Li et al. 2008).

There are no studies on the impact of traffic pollution on the morphological parts of the grasses. It was found that the lowest amount of heavy metals is accumulated in the seeds of plants, and the highest in their underground parts (Garcia and Milan 1998). In studies on cadmium and lead content in morphological parts of *Galega orientalis*, the higher lead content was observed in leaves, but in case of cadmium, it was observed in the shoots (Deska et al. 2011). The content of these elements in the studied plants is the sum of the amounts of the element absorbed by plants through the root system from the soil, taken in the form of fine dusts by stomata and accumulated in the tissue and the amount of dust deposited on the parts of plants (Aoyama and Kuroyanagis 1996). It should be noted that in the case of plants exposed to dusts containing heavy metals, the great importance is the dust settling on the surface of plant organs. It was proved the influence of surface cleaning of plants metals concentration on the change of their content in plants (Rossini Oliva and Mingorance 2006). Exact wash samples or remove the top layer of tissue resulting in a significant decrease of cadmium and lead content. Large amounts of lead and cadmium accumulate in the leaves of plants growing in areas with high air pollution by dusts containing these elements (Aoyama and Kuroyanagis 1996).

Accumulation of dust can be intensified by various features like surfaces covered with plant hairs, wrinkles, or a spongy or stubbly structure. These natural traps can significantly affect the permanent immobilization of molecules containing heavy metals. Grasses having leaf surfaces with varying degrees and covered with bristles, ciliated, or striated can retain the dusts (Hendry et al. 1992; Viard et al. 2004). Even larger traps can be grass inflorescences, which porous, composite structures can hold large quantities of dust. In this study, higher amounts of cadmium and lead in inflorescences of *D. glomerata* and *Alopecurus pratensis* are probably caused by these processes. The lower content of these elements in *Arrhenatherum elatius* results from the morphological characteristics of this plants species not favorable to the sedimentation of the dust. Additionally, this can affect the species characteristics related with the limited movement of the heavy metals from the soil.

The heavy metal content in the shoots is mainly related with the translocation of mobile forms of these elements taken from the soil and with their retention in the structure of the tissues. The obtained results indicate the importance of the species and the harvesting stage of grasses grown in areas with a higher degree of risk of dust sedimentation containing heavy metals. Proper selection of crop species may limit the risk of animal feeding. Regardless of the tested grass species, the most concentration of heavy metals was estimated in the plant materials which were collected at 1-m distance from the road. So, this part of grasslands should not be grazed by animals.

## Conclusions

1. The average lead content in the above parts of tested grass species *D. glomerata*, *Arrhenatherum elatius*, and *Alopecurus pratensis* amounted to 3.56, and for cadmium, it is 0.307 mg kg^−1^ DM, which indicated even lower values than the standard adopted for the feed using of grasses.
2. There were significant differences in the content of lead and cadmium in the studied species of grasses. *Alopecurus pratensis* has absorbed significantly higher amounts of lead (average of 4.11 mg kg^−1^ DM), while in *Arrhenatherum elatius* grass, a lower bioaccumulation of this elementary substance has been noted than in the other species (0.251 mg kg^−1^ DM).
3. Having analyzed the morphological parts of the grass species, we can observe that the lead and cadmium content in *D. glomerata*, *Arrhenatherum elatius*, and *Alopecurus pratensis* was significantly different. Cadmium concentration in the studied plants was different.
4. Distance from the road has significantly affected the contents of the studied metals in morphological parts of the grasses. The occurence is associated with a diversified structure of these plant organs.
5. The obtained results indicate a possibility to select species composition of grasses on areas threatened with heavy metal contamination of dust origin. The grass species recommended for sowing of land contaminated with lead is *D. glomerata*, and that contaminated with cadmium, *Alopecurus pratensis*
